# The third wave of China’s open door policy

**DOI:** 10.1007/s44216-022-00008-4

**Published:** 2022-10-18

**Authors:** Yongnian Zheng, Jie Li

**Affiliations:** The Institute for International Affairs, The Chinese University of Hong Kong, Shenzhen, China

**Keywords:** Third wave of opening, Unilateral opening, Internal and external circulation

## Abstract

In a world that is changing dramatically, China needs a third wave of opening. This article addresses several issues, including what the third opening looks like based on the two previous openings in modern China, why a third opening is necessary, and how to open up to the outside world in the future. In particular, a new period of strategic opportunity is required to solve the problem of “being scolded” and to achieve China’s sustainable development. This period will be created more proactively and unilaterally through five approaches, namely, (1) harmonizing domestic market standards, (2) reorganizing and re-identifying existing international rules, (3) modernizing the governance system, (4) allowing local and social level openness, and (5) promoting negotiations to join the Comprehensive and Progressive Agreement for Trans-Pacific Partnership (CPTPP) and advancing the U.S.–China Bilateral Investment Agreement negotiations. With respect to internal and external circulation, China can integrate domestic and international markets and internationalize China’s rules while aligning with the international community. Ultimately, the internationalization of China’s rules will be achieved through the integration of domestic and international markets.

In November 2020, China, ASEAN, and other countries signed the Regional Comprehensive Economic Partnership (RCEP). Completing the Comprehensive Agreement on Investment (CAI) negotiations between China and the European Union (E.U.) later the same year was another landmark event. On September 16, 2021, China formally applied to join the Comprehensive and Progressive Agreement for Trans-Pacific Partnership (CPTPP). These developments are significant in the history of China's contemporary opening-up, and they herald China’s third opening since the Opium War in 1840. In terms of the nature of the opening-up, while the first opening-up after the Opium War was forced and the opening-up that began in the 1980s was proactive, the current third opening-up is not only proactive but is also unilateral in many ways. In terms of the purpose of opening-up, the first opening-up was to survive and solve the problem of "being beaten" (“standing up”); the second opening-up was to solve the problem of "being starved" (becoming rich); and the third opening-up is to solve the problem of "being scolded" (becoming powerful), that is, by competing for international rule-making power, China seeks to move closer to the center of the world stage and play a role in global governance as a rising great power. This article begins with a brief review of the first two openings in Chinese contemporary history, then explains why a third unilateral opening is the solution to the problem of “being scolded” and concludes with five policy recommendations on unilateral opening.

## The two openings in modern China

In modern times, China has experienced two openings. Historically, the first forced opening was the result of the previous closed-door policy that had been in place since the Ming dynasty. During the Han, Tang, and Song dynasties, China was the most open country globally. However, starting from the Ming dynasty, China began to close itself and eventually fell behind. The end of the Ming and Qing dynasties deprived China of two related great eras, the Age of Oceans and the Age of Industrialization.

The Ming dynasty coincided with the beginning of the world's maritime era. From 1405 to 1433, Admiral Zheng He sailed seven times to Southeast Asia, the Indian subcontinent, West Asia and East Africa, demonstrating the great maritime power of the Ming Dynasty. The European maritime era began with the ventures of Portugal, followed by Spain, the Netherlands, England, and France. By any measure, the fleets of these European countries were not comparable with China's. However, many factors, such as the conservative ideology of the Ming dynasty, the problems of the northern frontier, and the court struggle, brought China's maritime development to a grinding halt. The Ming dynasty interrupted its state-organized maritime activities and banned private maritime activities. As a result, China’s maritime era ended.

The commercial revolution began first in Europe, followed by the industrial revolution, which led to the birth of the capitalist system. The rise of capitalism created great wealth for the West and led to world domination. While the budding of capitalism emerged during the Ming and Qing dynasties, China was never able to develop a capitalist system like the one in Europe. Although there is no single answer to the question, "Why did the industrial revolution first occur in Europe rather than in China," (North 1981) the Chinese imperial government's (particularly the Qing dynasty's) policy of seclusion was undoubtedly one of the most critical factors. In other words, its closed-door policy also deprived China of participating in the era of industrialization.

According to economic historian Angus Maddison, China remained the country with the world's largest GDP until the 1820s, accounting for one-third of the world's total GDP (Maddison 2007). However, 20 years later, the country was defeated by Britain in the First Opium War and again in the Second Opium War. After that, China became what Mao Zedong called a "semicolonial" country. Successive defeats in wars led Chinese political elites to realize that the strength of a country does not lie in its economic output, nor even in its military power alone, but in a new set of institutions, especially a political system.

However, this realization came at a price in blood. Only after China lost to Japan, viewed as its former student, did Chinese elites realize the importance of establishing a new institutional system. China's modern history is also the history of the Chinese political elite's search for a Chinese political system, from the political figures of the late Qing dynasty to Sun Yat-sen, Chiang Kai-shek, and Mao Zedong. With Mao Zedong at its core, the Chinese Communist Party (CCP) eventually found its way to success. The People's Republic of China was founded in 1949, ending successive defeats in modern times. Therefore, Mao Zedong's generation of Chinese Communists solved the problem of "being beaten".

The second period of opening began in the late 1970s and early 1980s. The Deng Xiaoping generation of leaders summed up the historical scale: "Closed will be backward, backward will be beaten". To avoid being beaten, China had to solve the problem of backwardness. Therefore, under Deng's leadership, China carried out a proactive policy of opening up to the world. China has seized almost every opportunity brought by globalization since the 1980s. Indeed, the expression "*jiyu*" ("opportunity") has been one of the most commonly used terms used by Chinese policy-makers since the 1980s.

Over the past 40 years, China has transformed itself from a "poor socialist" country to the world's second-largest economy and largest trading nation. In terms of GDP per capita, China has risen from less than $300 in the early 1980s to $12,000 today (The World Bank’s statistics 2021). An even more meaningful miracle lies in its eradication of poverty. China has lifted more than 800 million people out of poverty; 100 million people alone have emerged from poverty since the 18^th^ Communist Party Congress in 2012 (The China Daily 2018). China has announced that it has eradicated absolute poverty, a problem that has plagued the country for centuries (The State Council Information Office of the People's Republic of China 2021). Needless to say, the Chinese Communists of Deng Xiaoping's generation solved the problem of "being starved".

## The need for a third opening

In essence, for the current generation of CCP leadership, the third opening-up is needed to solve the problem of "being scolded." Why is China "scolded" by the international community, particularly the West? This question can be explained, at least in part, by the changing relationship between China and the West.

The relationship between China and the West has gone through several stages since the reform and opening-up. In the first phase in the 1980s, China adopted a policy of "*qing jinlai*" ("inviting in"). China needed capital for economic development, so it invited foreign capital to enter the Chinese market. At this stage, China had no substantial conflicts with Western countries. In the second stage in the 1990s, to join the World Trade Organization (WTO), China adopted a policy of "*jiegui*" ("convergence"); that is, China reformed its system of laws, regulations, and policies to comply with the rules of the global order, most of which the developed Western countries had established. At this stage, there was still no conflict between China and the West.

The third stage was when China started the "*zouchuqu*" ("going out") strategy after becoming a member of the WTO. Chinese goods went out first. After joining the WTO, China soon became the so-called "world’s manufacturing factory" (in fact, it was the "world’s assembly factory"), and a large number of products made or assembled in China were exported to the West and other parts of the world. This was followed by Chinese capital “going out.” China has proliferated and has quickly gone from a capital-starved economy to a capital-surplus economy. Like all other forms of capital, Chinese capital has begun to "go out", both state-owned and private. In recent years, Chinese technology has also begun to "go out". Although China has primarily been a technology-applying country rather than an originator of technology since its reform and opening-up, it has caught up with the West in some areas.

China's "going out" is in direct conflict with Western interests. Good quality, low-priced Chinese goods occupy an increasing share of the global market. Chinese capital has put pressure on Western vested interests. The West sees Chinese technology as a "challenge" or even a "threat" to its hegemony. Over the years, Western countries, especially the U.S., have focused their "complaints" and "abuse" against China on so-called "rules". The core idea of the West is that China has failed to comply with the "rules", such as WTO rules, investment and trade rules, freedom of navigation at sea, and human rights rules.

If "rules" are an essential source of "being scolded," then China needs to start with the "rules" to solve this problem. Only by mastering the power to make the rules will China solve the problem of "being scolded" entirely. Thus, China needs to open up for a third time. The third opening will be deeper than the previous openings intended to solve the problems of quantity and quality of investment and trade, technological upgrading and innovation; its intention is to assert China's voice and compete for rule-making power in the international arena, which is at the heart of international competition. It is even more so in the future relationship between China and the United States.

The third opening is not just a response to the problem of "being scolded" but also to China's sustainable development. An important lesson from the history of reform and opening-up over the past 40 years is that opening up to the world has often been the driving force behind domestic reforms. A popular view defines attracting foreign investment as the purpose of opening up, arguing that China already has enough capital of its own and does not need foreign investment. However, this view is faulted and not evidence-based. There are multiple rational considerations why China must still vigorously introduce foreign capital even when it has sufficient capital.

First, China has valued the more advanced management experience and better international rules that foreign investors brought with them than what was available domestically. To a large extent, it was using the pressure of international rules to strengthen the momentum of domestic reform, allowing China's rules to "approach" those of the West on its initiative. With the gradual upgrading of China's industrial economy and the emergence of new business models, China has begun to have rule-making capabilities similar to those of the West in certain areas.

Second, pressure from international sources is growing in terms of rules, and China has to open up and make its voice heard for its further development. On February 19, 2021, while attending the Munich Security Conference, European Commission President von der Leyen invited the United States to join the E.U. initiative to regulate digital markets and create rules for the digital economy together. "Together, we can create digital economy rules that work globally: a set of rules based on our values, people and diversity, inclusion and protection of privacy," said von der Leyen (Reuters 2021). The fact that Europe lacks high-tech companies like those of China, but has spared no effort in competing for digital rules, shows the importance of the competitiveness of "rules."

In the internet and digital age, the West's concern about China derives from its ability to make rules. Recently, Western think tanks have focused on the issue of "technology standards" and linked it to U.S.–China relations. They argue that China is devoting more resources to developing technology standards and suggest that the U.S. and European countries cooperate more to maintain a long-term advantage (Klein 2020 ). Western think tanks generally agree that technology standards have become a central issue in the competition between major powers. The U.S. should become more actively involved and seek to "squeeze out" other powers in critical technology areas.

According to an article published by the Woodrow Wilson International Center for Scholars, technology standards have taken center stage in discussions around U.S. national security and the evolving dynamics of great power competition (Girard 2021). The 2020 Annual Report of the U.S.–China Economic and Security Review Commission analyses the U.S.–China global strategic relationship, economic and trade relations, political diplomacy, and security, accusing China of "competing" with the West for the control of crucial technology standards and attacking China's use of technology standards as a "policy tool" for economic and geopolitical interests (US-China Economic and Security Review Commission 2020). The Center for a New American Security recently released a report saying that technology is critical to national economic power and security and is a central issue in the competition in U.S.–China relations. The report argues that the U.S. should become more active in developing international technology standards (Rasser 2021). It is clear that the West has realized the need and urgency of engaging with China on rule-making power in the new digital economy, and China should respond by accelerating its pace accordingly.

Third, and more importantly, given that the diffusion of technology would be impossible without the flow of capital, China must open itself up to foreign capital. It is indisputable that most of the original technologies of modern times have come from the West. In recent years, global competition in science and technology has entered a period of restructuring, with economic and trade frictions between the U.S. and China and intensified competition in science and technology, as well as a "wave of counterglobalization," all posing new challenges to international cooperation in science and technology.

Some believe that China can invent whatever (technology) is blocked by the U.S. However, there is no scientific basis for this view. All countries want to develop technology. However, the invention of new technology requires much investment and time. Even significant investments do not necessarily lead to new inventions in the end. The road to new technologies is always fraught with significant risks. This is not to say independent invention is unimportant. However, independent innovation should be carried out in an open environment, never behind closed doors. China should not be under the illusion that the development of original technology will succeed in the short term through complete self-reliance.

The U.S. technology embargo has given China a new "task list" for its innovations (Kharpal 2021). However, China needs to precisely identify these "tasks" and see which ones it should further open up to foreign (especially non-U.S. developed) capital, technology, and human resources. This step will shorten the time and reduce the cost of technological innovation. At present, the Chinese government's new "external circulation" strategy is not only the circulation of capital and trade but, more importantly, the circulation of technology, especially core technology.

Historically, the essence of technological progress is openness; if a country is closed, technology will decline. China's history teaches the same lesson. China is right to emphasize a new system of statehood to develop core technologies, but the system of statehood must be an open one. The Soviet Union had rapid technological progress, but it was not open to competition from the outside, and it quickly declined.

In terms of the laws of development of science and technology itself, scientific and technological innovation also requires international cooperation and the joint participation of the international scientific community. Despite the recent deterioration in U.S.–China relations, the Western scientific community (including that in the U.S.) still has a positive attitude toward international cooperation. The current pandemic has been continually politicized and stigmatized by populism in various countries. However, the Western scientific community still holds a high degree of approval of China's initiatives to combat the pandemic and actively cooperates with the Chinese scientific community. On December 18, 2020, Li Xiaohong, President of the Chinese Academy of Engineering, Sir Jim McDonald, President of the Royal Academy of Engineering, and John L. Anderson, President of the U.S. Academy of Engineering, signed "The NAE, CAE, and RAE Joint Statement on COVID-19." The statement advocates that international collaboration, not competition, should be the driving force for progress in device development, diagnostics, vaccine development and production, and data analysis in response to the pandemic (National Academy of Engineering 2021).

The U.S. government has increased its scrutiny of Chinese high-tech companies in the name of protecting national security and has worked to promote technological "decoupling" from China. However, Western companies, including U.S. firms, have been trying to circumvent the ban and continue to "link up" with China. In October 2020, the Dutch company ASML said that its exports of DUV (deep ultraviolet) lithography to China would not be subject to U.S. restrictions or require U.S. approval. Although Huawei is more desperate for EUV (extreme ultraviolet) lithography than DUV, this already shows that a complete embargo is difficult to achieve in the international high-tech sector (Nellis 2021). However, international cooperation demands an open China. Once China is closed, then no international cooperation is possible. China can only attract more companies and academic communities to strengthen its technological cooperation by being more open.

## The creation of a new strategic opportunity

It is time to create a new type of "strategic opportunity." Chinese leaders used to talk about "international opportunities (*guoji jiyu*)," but the international opportunities of the past and those of today are entirely different concepts. Previous international opportunities were reactive; they emerged from the external context and China rushed to seize them. However, if Chinese decision-makers continue to think of international opportunities the same way, they will likely make errors. Today, China is able to create many international opportunities on its own rather than waiting for others to give it opportunities.

To create a new period of strategic opportunity, China has to open up to the outside world more proactively, even unilaterally, which is the central theme of the third opening. A unilateral opening-up means that China must open up to the U.S. (the West) even if the U.S. (the West) does not open up to China. Historically, the British Empire was much more successful than the United States. In specific periods, the British Empire was unilaterally open; in contrast, the United States always followed an approach of reciprocal openness, that is, engaging with other countries only if they developed relations with the United States. Unilateral openness was more effective than reciprocal openness. When Napoleon tried to defeat Britain commercially with his "continental blockade", Britain ultimately defeated Napoleon's blockade pressure with a "unilateral opening" and gained more breakthroughs in trade.

In recent years, China has faced increasingly greater pressure and challenges from the U.S.-led West in creating a period of new strategic opportunities. According to a report in *The Wall Street Journal* in early February 2021, the Biden administration's crackdown on China in the technology sector is comparable to that of the Trump administration (McKinnon and Leary 2021). The Committee on Foreign Investment in the United States (CFIUS), whose National Security Task Force closely monitors and scrutinizes U.S. start-ups for any ties to China-related investors, will play a vital role (Somerville 2021).

On February 24, 2021, President Biden officially signed an executive order for a 100-day review of the supply chains of four essential products: semiconductor chips, high-capacity batteries for electric vehicles, rare earth metals, and pharmaceuticals. The order also guides six sectoral reviews modeled on procedures used by the U.S. Department of Defense to strengthen the industrial base in defense, public health, communications technology, transportation, energy, and food production (The White House 2021).

The intention of these initiatives to point to China is evident. The Biden administration has even defied the opposition of the U.S. business community by putting into effect a sweeping Trump-era rule to counter the Chinese technology threat. The rule would give the U.S. Department of Commerce the power to ban technology and business transactions considered so-called national security threats to protect the U.S. supply chain (McKinnon 2021). The National Security Commission on Artificial Intelligence, led by former Google executive director Eric Schmidt, made several recommendations in its Final Report to the U.S. Congress on A.I. competition with China and the semiconductor supply chain to curb and contain China's progress in A.I (National Security Commission on Artificial Intelligence 2021).

The Biden administration also formed a proposed "Summit for Democracy", which seeks to establish straightforward programs to counter China (U.S. Department of State 2021). For example, the administration attempts to organize a small group of democratic countries to address specific advanced communications and artificial intelligence issues. With other critical democracies, the U.S. intends to form a technology alliance to develop new telecommunications technologies to reduce reliance on Huawei's 5G equipment. The administration will also put forward several proposals to block the sale of advanced semiconductor manufacturing technology to China, which U.S., Japanese and Dutch companies dominate, to maintain a several-generation lead over Chinese semiconductor manufacturing technology. Senate Majority Leader Schumer said that he had instructed lawmakers to draft a bill to limit China's rise. The bill, which could amount to $100 billion, targets artificial intelligence, quantum computing, and semiconductors (Stein and Whalen 2021).

The U.S. is increasingly worried about China's rise and will do everything possible to suppress China. China should have no illusions in this regard. However, China must resort to reason to deal with this situation. The "unilateral" opening referred to here is the product of reason. China is also strong enough to open up unilaterally. After over 40 years, China has accumulated enough material wealth and experience to deal with the adverse effects of globalization and the external pressures exerted by the U.S. (and the West).

China's comparative advantage or strength is not its "sparring" with the U.S. but rather its potential for opening up and its market size. Over the years, China has been working toward an opening that is both deeper and broader. However, China has not opened up enough in some areas. Given the potential for opening up plus the size of the market, opening up in any one area would be enough to change the flow of international capital.

The unilateral opening of an economy with a middle-class market of 400 million people will attract increasing Western capital to China. On January 24, 2021, the United Nations Conference on Trade and Development released a report that indicates China had replaced the U.S. as the world's largest foreign direct investment (FDI) recipient in 2020, attracting $163 billion in inflows, having taken over the number one position for decades (Reuters 2021). With the global economic shocks and uncertainties caused by the epidemic, the Chinese market has become a "ballast" for the global economy and a key market for many multinational companies to grow in 2020. Indeed, the three largest luxury car brands, BBA (Mercedes-Benz, BMW, and Audi), achieved significant sales growth in China in 2020, setting their best sales records since entering the Chinese market (Fukao 2021).

After Trump launched the U.S.–China trade war, the U.S. administration tried to use fiscal funds to attract U.S. capital away from China and back to the U.S. At the time, the Abe government in Japan also introduced similar policies to attract Japanese capital. However, none of these had a visible effect. A Goldman Sachs report shows that most U.S. companies in the semiconductor equipment, materials, and health care sectors are not only not moving out of China but are expanding their production in the country (Anonymous 2020). The Japanese government's special funding introduced in April 2020 to finance the return of Japanese companies has not worked as intended, with only 5% of Japanese companies applying for subsidies. Of those that have, the main aim is not to withdraw from the Chinese market but to take the opportunity to restructure, according to the Japanese media JBpress News (Anonymous 2020). According to a survey by HSBC Qianhai Securities, nearly two-thirds of the world's more than 900 institutional investors and large companies plan to increase their investment in China by 25% in 2021 (https://www.hsbcqh.com.cn/-/media/gbm/gbm-jv/solution/hsbc-qianhai-securities-opportunities-for-international-investors.pdf). Regardless of the position of the U.S. administration or any Western government, Western capital, including the U.S., will not abandon the vast Chinese market.

The U.S. is trying to divide China. It is also trying to form a "united front" with its allies against China in various forms of "alliances among democratic states." What should China do? China must respond to U.S. political and ideological means (i.e., democratic institutions and liberal values) with economic and capital means (i.e., openness and markets). In short, a third opening is the most effective way for China to prevent the U.S. from forming a "united front" against China. As long as China is open and as long as the U.S. (and the West) remains a capitalist state, China and the U.S. will not decouple entirely.

## Policy recommendations on the unilateral opening

China needs to think about how it can reshape the rules of the world market by opening up for the third time. It can make a difference in at least the following areas.

1. *Harmonizing domestic market standards and constructing nongovernment-dominated rules to avoid restricted radiation diffusion of rules and policy arbitrage by foreign investors.*

The third opening-up must emphasize the importance of rules. There are technical rules and rules for all aspects of society, investment, and trade in the rules. First, it is necessary to unify the different rules in different parts of the country. Modern states are more potent than traditional states because the former have uniform rules, whereas the latter do not. Modern sovereign states first rose in Europe, and sovereign states are sovereign because they have uniform rules. After World War II, European states expanded from having internal rules to rules between states, forming the European Union. The E.U. is strong because it has a uniform set of rules. Similarly, various other regional organizations, including NAFTA, the ASEAN Free Trade Area, and RCEP, are all embodied in shared rules.

Today, China advocates a "dual circulation" approach to sustainable development, which can be interpreted from different perspectives. However, by unifying domestic rules through "dual circulation" and internationalizing them, China can achieve sustainable development internally and further integrate into the international economy, enhancing its rule-making power or voice in international affairs.

There is a need to facilitate general internal circulation through harmonizing rules. Uniform rules are essential to both the market economy and the rule of law. The essence of a market economy is the rule of law, and the harmonization of rules is a prerequisite for the rule of law. If the rules are unharmonized, it is difficult for the economies to achieve radiative diffusion.

There are no uniform rules within the various economic regions of the Beijing-Tianjin-Hebei region, the Yangtze River Delta and the Pearl River Delta, among the provinces or different cities, or among different enterprises. Each offers ideal conditions to foreign businesses, apparently competing for "openness" but, in fact, moving toward closure. The rules of doing business for foreigners are very different from one place to another, even between two industrial parks in the same district, making it difficult for foreigners to choose and settle. Internal rules must be harmonized across China or at least dovetailed. The absence of unified rules also means that China does not yet have a unified market, without which it is difficult to translate China's total trade into rules. The harmonizing of internal rules is the only way for China to strengthen its internal competitiveness and achieve better access to the world.

At the same time, inconsistent rules can easily lead to "unhealthy competition" between different regions and enterprises to attract foreign investment or even to unrealistic offers of unsuitable local conditions to attract foreign investment. In the early days of the reform and opening-up, some places went to great lengths to lower the standards of local environmental and labor protection policies, which led to economic development in the short term at the expense of the social and political interests in long-term development. Foreign companies also put themselves in a favorable bargaining position by using the varying rules between different regions to negotiate with localities. Compared to the early days of reform and opening-up, China's standards and requirements for attracting foreign investment have increased significantly. It is more important to create better synergies and stricter rules to attract high-quality foreign investment.

The absence of rules or the lack of uniformity in rules affects the efficiency and effectiveness of Chinese capital invested internationally. State-owned enterprises (SOEs) have played a pioneering role in the Belt and Road Initiative, but there are no rules for competition between SOEs. Often, two or more SOEs compete viciously in foreign countries, damaging the country's interests and causing a negative impact on the local country. Competition between state-owned and private enterprises is even less regulated, resulting in substantial economic losses.

Furthermore, after harmonizing rules at the governmental level, it is also necessary to focus on harmonizing rules at the social (market) level. If there are no rules for effective industry organizations at the social (market) level, government rules will be null and void. Even if there is no vicious competition among local governments, it will be challenging to avoid vicious competition among enterprises and industries that lower their own rules.

In establishing market rules, China will have to learn from the relevant experience in Europe and the United States and establish rules led by non-governmental organizations. For example, the British Retail Consortium (BRC), the American Society for Testing and Materials (ASTM), and the Institute of Electrical and Electronics Engineers (IEEE) have blocked Chinese companies and products based on their rules and standards. The purpose of this learning is not to construct targeted barriers but to provide Chinese companies and industry organizations with more tools for gaming. The "two legs" of government and industry must work together to construct a rule structure that is more conducive to the influence of Chinese rules.

2. *Reorganizing and re-identifying existing international rules, influence, reform, and even creating new international rules.*

China faces several vital issues and challenges when competing with the West in terms of rules. The first is reforming the existing rules. The world system is not abstract but has various rules. Many of the existing rules are under the domination of the U.S. (the West). They reflect the interests of the West rather than those of developing countries, including China. China does not want to be a revolutionary in the international community. However, China is a reformer and advocates reform of the international system and rules to make the world system more just and equitable. As China's voice increases, it will need to differentiate between international rules detrimental to itself and international efficiency while using better international rules to promote domestic reform.

Furthermore, China should turn the advantages of the market into the advantages of the rules and make good use of the rules as a "buffer zone" for dealing with external relations. The Western dominance of world rules and Western markets are inseparable. China is the second-largest economy and the largest trading country, but it still does not have the power to make rules. In the 1980s, China introduced a "market for technology" policy, first applied to the automotive industry. Under this policy, Chinese car companies learned the advanced technologies and management approaches of the West. Although this "market for technology" has helped China's economic development to a certain extent, in the longer term, the "market for technology" strategy has not been a success. For example, in 2003, BMW Brilliance was established as a joint venture between BMW of Germany and Brilliance Group. On November 20, 2020, Brilliance Group, which had no core technology and lacked brand influence, was officially bankrupt (Anonymous 2021). Brilliance's dismal exit shows that China's manufacturing industry's "market for technology" path is blocked.

The third opening-up should be a shift from "market for technology" to "market for rules", especially in the new sector of the digital economy. China needs to learn from European countries that are good at making rules. In terms of internet development and rules, even though European countries do not have large internet companies, they actively use their market power to develop internet rules. The E.U. has introduced many internet regulatory laws and regulations, competing with the U.S. and China in rule-making.

Despite having many large internet companies and a large share of the world's internet, China has little to no rule-making power. Most Chinese internet companies have developed within the Chinese market and have rarely "gone global". Their business models were from the U.S., so they have inevitably followed the U.S. scheme in terms of rules. The development of China's internet industry is not strong enough to attract sufficient attention, and the problems encountered are the same as those encountered by American internet companies. However, the U.S. crackdown on Huawei and ByteDance has signaled a warning that if China does not have enough rule-making power and continues to "Americanize" the rules, its future high-tech companies will struggle to thrive.

Despite its solid geopolitical and ideological considerations, the U.S. still uses "rules" (including laws) to crack down on Chinese technology companies. In contrast, lacking effective rule-based countermeasures, China’s response tends to be more overtly political. It cannot use rules-based means to "package" its objectives as the U.S. does and is often more passive in its diplomatic games with the West. This issue is evident in the Huawei and Meng Wanzhou incidents. In the absence of a rules-based approach as a buffer zone, the two incidents escalated directly into a "hard" conflict at the diplomatic level between China, Canada, the U.S., and even China and the West, making it more difficult to resolve and back down. Had there been rules in place, the incidents themselves could have been "softer." It has not been long since China introduced the "Rules on Counteracting Unjustified Extraterritorial Application of Foreign Legislation and Other Measures," which is a good start to counteract through the leverage of rules in the face of unreasonable U.S. (Western) suppression (Ministry of Commerce of the People's Republic of China 2021).

China must also take the initiative in creating and participating in formulating future rules. In recent years, by promoting economic regionalization and globalization, China has taken the initiative to create some regional or global rules, such as the "Belt and Road Initiative," the "Asian Infrastructure Investment Bank", and the "BRICS Bank". The next step for China is to consider attracting more countries to join and getting more countries to accept its rules.

As a great power, China should not learn from the West of Old and impose the rules it makes on other countries; instead, it needs to take the interests and needs of other countries fully into account when making rules. China needs to pay more attention to international cooperation when making rules, especially with non-Western developed countries, and needs to learn from the mistakes of the U.S. when using its global economic leadership and focus on long-term interests.

China can explicitly declare that it has been a victim of the unilateral rules of the U.S. (West). Today, China's rules will necessarily present more inclusiveness and pluralism, creating a more open system of rules. In the field of the internet, where China already has a vibrant practice, it should summarize and generalize to promote the formulation and creation of international rules for the internet and the multi-polarization of international internet rules.

3. *Modernizing the national governance system and improving governance capacity.*

The opening-up of the past was more about pushing domestic reforms. The third opening is about achieving higher-quality domestic governance, i.e., modernizing the national governance system and capacity.

During the four years of the Trump administration, the concept of "reciprocity" formed the cornerstone of his policy toward China, and bilateral relations fell to an all-time low. Before Trump was elected president in 2016, the principle of "reciprocity" was generally applied only in investment and trade. The U.S. believed that China had failed to deliver on its promise to open up investment and trade over the past 20 years.

The Trump administration took full advantage of such grievances and complaints by highlighting the "reciprocity principle" in its dealings with China. The administration imposed restrictions on Chinese diplomats and journalists and revoked visas for students and scholars associated with the Chinese People's Liberation Army. Former U.S. Treasury Secretary Paulson has long been friendly to China and supportive of greater cooperation and interaction between the U.S. and China. However, in a speech on U.S.–China relations last November, he called for increased "targeted reciprocity" between the two sides (Fromer 2020). The Biden administration is no different. The "reciprocity principle" will become increasingly important in China's future relations with the U.S. and other Western countries.

However, the restrictive access in some regions to opening up results from China's failure to meet its domestic governance capacity requirements, i.e., the so-called "unmanageable" governance or "poor management". This problem is particularly true in the internet sector and is certainly not limited to it. As the competition between the U.S. and China in technology is intensifying, the "principle of reciprocity" will become more evident in high technology. Most U.S. internet companies, especially those involved in ideological issues, cannot enter mainland China.

In the long run, opening up the orderly entry of American internet companies (or the European internet companies) into mainland China is a real problem that is difficult to avoid. The essence of the problem is how to manage these companies effectively without affecting political, economic and social stability. China needs a set of internet management rules that are based on Chinese characteristics and, at the same time, comply with international internet management rules.

At present, countries worldwide are strengthening their governance of social media. These countries include developed countries in Europe and the United States and many countries lagging in the internet field, as social media has profoundly impacted these countries' political and social stability. China can learn from and refer to some of the experiences of other countries to improve the rule of law and institutionalization of internet content management.

4. *Greater focus on local and social openness allows for finer granularity and more multidimensional levels of openness.*

The third opening is unilateral in many areas. As mentioned earlier, the primary purpose of the unilateral opening is to prevent the Western world—which is not internally ironclad—from forming a united front against China in the face of vicious international competition. There are two paths of opening up available.

First, openness and exchanges should be promoted at the local level. China–U.S. friendship provinces (states) and cities have reached 50 and 231 pairs, respectively. Lan Jin, president of the Oregon China Council, said in an interview with the Xinhua Press that "the diversity of subjects in local and private interactions is particularly helpful in building a firm foundation of public opinion for friendship and mutual trust." (Anonymous 2021). While the Trump administration intensified its trade war against China and decoupled various fields, local governments, including California, are increasing their cooperation with China. Many developed municipalities in China have the advantage of kinship with immigrants and docking of industries in opening to the outside world. This phenomenon is especially true in cities in Fujian, Zhejiang, and Guangdong, where the need for opening up is more pressing and has more local characteristics. These places can be encouraged to open up and be more grounded and closer to the people.

Second, openness and exchanges should be promoted at the societal level. As China's economy grows further and the diversity of social interests increases, the needs of different social groups and social classes to open up are becoming more diverse. At the same time, the power of various social groups to hold together in their interests to carry out external exchanges is also increasing. Opening up at the social level is far more precise and effective than opening up at the governmental level, allowing for a more prosperous network of openness and more open nodes. As mentioned above, large Western corporations and Wall Street capital have not abandoned the Chinese market; in contrast, they are bullish on China's huge market and stable business environment and are adding to their positions in the country in a large way. The latest China-World Economic Dependence Index compiled by the McKinsey Global Institute shows that China's dependence on the world economy has declined relatively in trade, technology, and capital. Conversely, the world's dependence on the Chinese economy has increased relatively (Woetzel et al. 2019).

5. *Actively promoting negotiations to join the CPTPP and advancing the U.S.–China Bilateral Investment Agreement negotiations.*

Behind the rules are standards. Shortly after joining the RCEP, China applied to join the CPTPP. The RCEP and CPTPP are different. The former is mainly about traditional investment and trade tariffs, while the latter is about standards. Competition for standards is conducive to improving China's technology and the quality of its products. At the end of last year, China also completed negotiations with European countries on the China-EU agreement. These results show China's strong willingness to participate in developing future standards.

These new developments have helped activate the U.S.–China Bilateral Investment Agreement (BIT) negotiations. Prior to the launch of the CEIBS negotiations, the U.S. and China launched BIT negotiations in 2008, which lasted seven years and 34 rounds of negotiations by 2015. After Trump took office, the U.S.–China BIT negotiations were on hold. With the Biden administration in power, there is a possibility of resuming the BIT negotiations between the U.S. and China. The successful completion of the China-EU investment agreement negotiations also allows activating the U.S.–China BIT negotiations by improving the quality and shortening the length of the negative list already exchanged and reaching an early bilateral investment agreement. Even if the U.S. is not yet ready to reopen negotiations, China can display its goodwill.

## Conclusions

If competition between China and the U.S. is inevitable, China should see the competition as an opportunity to open up further. Historically, whichever country is more open can develop, while a closed country will eventually decline no matter how powerful it is. The reasoning is simple. It is only in a state of openness that world markets exist, and elements of production flow to those economies that are open.

The next step between China and the U.S. is technology competition and, more importantly, rules competition. If concluded, a U.S.–China BIT could allow the U.S. side to better understand China's demands and attitudes on rule-making. The U.S. may gradually accept the corresponding Chinese demands through negotiations and find a new platform and arena for rule competition between the two sides instead of the Trump-era sanction initiatives.

China's negotiations with the E.U. have demonstrated its capability to conduct rule-based negotiations. China succeeded in making its own market-based rules, which are acceptable to developed countries. If a similar agreement can be reached with the United States, China can further strengthen its ability to externalize its rules. China will truly move from benchmarking developed country standards to becoming a significant player in international rule-making.

To summarize, the core of the "internal circulation" is the unification of domestic rules, and the "external circulation" is the internationalization of domestic rules. If China sees the third opening-up in terms of the development of rules and standards, it can raise the profile of its opening-up. The third opening should be a broader, deeper and higher level of opening. By connecting the internal and external circulation, China can integrate domestic and international markets and realize the internationalization of China's rules while aligning with the international community. Only in this way can China simultaneously achieve sustainable internal development and a natural rise in the external world.

## Data Availability

Not applicable
